# Soil Biota Reduce Allelopathic Effects of the Invasive *Eupatorium adenophorum*

**DOI:** 10.1371/journal.pone.0025393

**Published:** 2011-09-28

**Authors:** Xunzhi Zhu, Jintun Zhang, Keping Ma

**Affiliations:** 1 State Key Laboratory of Vegetation and Environmental Change, Institute of Botany, Chinese Academy of Sciences, Beijing, China; 2 College of Life Sciences, Beijing Normal University, Beijing, China; Freie Universität Berlin, Germany

## Abstract

Allelopathy has been hypothesized to play a role in exotic plant invasions, and study of this process can improve our understanding of how direct and indirect plant interactions influence plant community organization and ecosystem functioning. However, allelopathic effects can be highly conditional. For example allelopathic effects demonstrated in vivo can be difficult to demonstrate in field soils. Here we tested phytotoxicity of *Eupatorium adenophorum* (croftonweed), one of the most destructive exotic species in China, to a native plant species *Brassica rapa* both in sand and in native soil. Our results suggested that natural soils from different invaded habitats alleviated or eliminated the efficacy of potential allelochemicals relative to sand cultures. When that soil is sterilized, the allelopathic effects returned; suggesting that soil biota were responsible for the reduced phytotoxicity in natural soils. Neither of the two allelopathic compounds (9-Oxo-10,11-dehydroageraphorone and 9b-Hydroxyageraphorone) of *E. adenophorum* could be found in natural soils infested by the invader, and when those compounds were added to the soils as leachates, they showed substantial degradation after 24 hours in natural soils but not in sand. Our findings emphasize that soil biota can reduce the allelopathic effects of invaders on other plants, and therefore can reduce community invasibility. These results also suggest that soil biota may have stronger or weaker effects on allelopathic interactions depending on how allelochemicals are delivered.

## Introduction

Exotic plant invasions can have substantial impacts on ecosystem structure and on human economic systems [1]. Invasive plant species have altered biodiversity, functioning of natural systems, and aesthetic value of many habitats around the world [2], [3]. Multiple hypotheses have been put forward to explain the remarkable success of many exotic invasive species [4]. The Novel Weapons Hypothesis [5], [6], which was initially based on the study of diffuse knapweed (*Centaurea diffusa*) and spotted knapweed (*C. maculosa*), argues that invaders may possess novel chemicals that are more phytotoxic to naïve, native plants in the invaded range than to adapted species in the invader's native range [7], [8]. However, with regard to knapweed allelopathy, the inhibitory effects of the putative allelochemical catechin are not always observed in experiments conducted in natural soil or in the field [9], and the debate is ongoing [10]–[13]. Allelopathic effects are largely determined by chemical, physical, and microbial components of soil that determine the fate of allelochemicals in the environment [14]. Initial reports of consistent soil catechin concentrations have been shown to be highly inaccurate [15]. Further, there has recently been a correction and clarification of the Bais *et al.* 2003 Science paper, stating that the high and consistent levels of allelochemical in soils that were originally reported had been irreproducible. Currently, extractible catechin is thought to decline rapidly in field soil through chemical degradation, binding to clay and organic matter, microbial degradation or a combination of the three.

Soil is one of the most important factors contributing to the “invasibility” of natural habitats, and may prevent or facilitate the establishment and spread of exotic invasive species [2], [16]. Soil biota can have substantial effects on interactions between invasive and native species [17], [18]. Some evidence suggests that soil biota in some invaded ecosystems may facilitate exotic invasion [19], [20]. However, it is possible that soil biota may also reduce the potential for novel biochemical interactions between invasive and native plants. To date, allelopathy research on invasive species has focused mainly on phytotoxins that cause direct interspecific interference between invaders and resident plant species. Plant-soil feedback processes need to be taken into consideration in the novel weapons hypothesis, and links between novel biochemistry and soil microbial communities need to be clarified.

The invasion by *Eupatorium adenophorum* Spreng. (croftonweed) in China is one of the most dramatic examples of the replacement of native vegetation by exotic plant species [21]. This exotic species has long been suspected of having allelopathic effects on resident native plants in its invaded range [22]. Baruah *et al.* [23] demonstrated that a chloroform extract of the aerial parts of *E. adenophorum* inhibited germination and seedling growth of *Allium cepa*, *Raphanus sativus*, and *Cucumi sativus*. Song *et al.* [24] argued that water-soluble allelochemicals of *E. adenophorum* could have allelopathic effects through natural leaching from leaves by rain. Many studies have indicated that aqueous leachates from *E. adenophorum*, particularly from leaves, significantly inhibit germination and seedling growth of other plant species such as *Brassica rapa* [25], *Chloris gayana, Ixeridium gracile* and *Mariscus cyperinus* [26]. In recent years, twelve organic compounds, including tartaric acid dimenthyl ester, cyclohexanamine N-cyclohexyl and diethylene glycol dibenzoate, have been extracted, isolated and identified with GC-MS from aqueous leachates of *E. adenophorum* [27]. Two compounds identified with chromatographic fractionating and bioassay-guided methods and NMR techniques are considered the primary phytotoxins: 9-Oxo-10,11-dehydroageraphorone [4,7-dimethyl-1-(propan-2-ylidene)-1,4,4a,8a-tetrahydronaphthalene-2,6(1H,7H)-dione] and 9β-Hydroxyageraphorone [6-hydroxy-5-isopropyl-3,8-dimethyl-4a,5,6,7,8,8a-hexahydronaphthalen-2(1H)-one] [28], [29].

Previous allelopathic studies of *E. adenophorum* have been based mainly on laboratory bioassays and have not taken natural conditions of different habitats such as soil into consideration. Therefore, it remains uncertain whether allelopathy contributes to *E. adenophorum* invasion [30]-[33]. In fact, some studies have found that the addition of activated carbon to soils from *E. adenophorum'*s invaded range did not ameliorate the negative effects of *E. adenophorum* [34], [35]. To understand the role of allelopathy in *E. adenophorum* invasion in the field, we: (1) examined whether soils from invaded fields influenced allelopathic effects of aqueous extracts from *E. adenophorum* leaves, (2) explored whether soil microbes were responsible for the effects of the soils on *E. adenophorum* allelopathy, and (3) evaluated whether *E. adenophorum* allelochemicals degraded rapidly in invaded field soils.

## Materials and Methods

### Site description

In May 2007, three field sites (20 m×30 m) were established in Panzhihua, in the Hengduan Mountain range in Southwest China. Detailed descriptions of the study sites and soil conditions can be found in our group's previous papers [25], [31]. The study area (101°08′-102°15′E, 26°05′-27°21′N) is characterized by a subtropical hot and arid valley climate with pronounced wet and dry seasons. Rainfall is mainly concentrated in July, August and September. The three sites had similar elevation, topography, gradient and soil types, and were invaded by *E. adenophorum* approximately 15–20 years ago. However, plant community composition differed among the three sites (Table 1). Site I, an evergreen broad-leaved forest, was dominated by *Machilus pingii*, *Cyclobalanopsis plaucoides* and *Lithocarpus dealbatus*, with a very low density of *E. adenophorum*. Site II was a deciduous broad-leaved forest, with *Alnus cremastogyne* and *Camellia oleifera* as the dominant species in the tree layer, and a dense *E. adenophorum* population in the understory layer. Site III was along a roadside with no trees or shrubs and with an extremely high density of *E. adenophorum*. In each site, four 1m×1m plots were randomly chosen and the height, percent cover and fresh weight of *E. adenophorum* were recorded (Table 1).

**Table 1:** Characteristics of the three field study sites.

sites	*E. adenophorum* height (m)	*E. adenophorum* cover (%)	*E. adenophorum* biomass (kg/m^2^) (Mean±S.E.)	Tree cover (%)	Shrub cover (%)	Herb cover (%)
Evergreen broad-leaved forest	0.2-0.4	10	0.16±0.06	90	30	60
Deciduous broad-leaved forest	0.4-0.8	60	2.75±0.42	60	10	80
Roadside	1.2-1.6	90	7.26±0.70	0	0	95

### Soil experiment

To assess whether natural soils influence interspecific allelopathic effects, we conducted a soil experiment using *Brassica rapa* as a receptor plant species in May 2007, since *E. adenophorum* is actively growing and likely releasing more allelochemicals in late spring [27]. In each 1 m x 1m plot at the study sites, the vegetation and litter on the soil surface were removed, and then the whole soil from 0–10 cm in depth was collected. Fresh soil was immediately sieved (2 mm) to remove roots and gravel. Then the soil was used to fill 24 1370-ml pots (upper diameter 14 cm, lower diameter 10 cm, height 12 cm), with 1 kg of fresh soil in each pot, and two pots for each plot at each site. Another eight pots were filled with sand. At the same time, fresh *E. adenophorum* leaves were collected from a natural population outside of, but not far from, the three field sites. The leaves were immediately extracted with distilled water (1 g fresh material: 10 g distilled water) for 36 h at 25°C (improved on [27], [28]). Then the leaves were removed and the aqueous leachate was filtered (75 mm glass funnel with one sheet of 90 mm folded filter paper). Seeds of *B. rapa* were purchased from Panzhihua Vegetable Market. Thirty *B. rapa* seeds were planted 1–2 mm below the surface in each pot for germination trials. Then 200 ml of leachate were poured evenly across the surface of the soil in half of the pots. The remaining pots were used as controls and treated with equivalent volumes of distilled water. In total, 16 pots received the leachate treatment, and 16 pots were treated with distilled water. The experiment was replicated four times, using the four plots within each site as replicates. The experiment was conducted in a greenhouse at Panzhihua University campus, where the environmental conditions such as average temperature (30°C at daytime and 25°C at night) and light (10-h-light/14-h-dark photoperiod, using mercury lamps) were similar to where *E. adenophorum* and native species occur. Distilled water was administered to maintain a constant moisture regime of *c.* 50% field soil water capacity. All pots were randomly arranged. Germinated seeds of *B. rapa* were counted after 10 days incubation, and then whole fresh seedlings (including shoots and roots) were excavated from soil, washed with distilled water and weighed. All data were normally distributed. Two-way ANOVAs were conducted on percent germination and the total biomass of all seedlings in each pot with soil source and leachate treatment as fixed factors.

### Sterilization experiment

To explore whether soil microbes were a key factor explaining the differences in allelopathy among soils, we repeated the soil experiment in July 2007 with both non-sterile and sterilized soils. Methods were the same as for the soil experiment, except as described below. In total, there were 24 pots with natural soil (3 sites × 4 plots × 2 leachate treatments) and another 24 pots with sterile soil. Soils were collected from the three field sites as described above. One half of each soil was sterilized by triple autoclaving (121°C, 0.105 MPa, 3 h/cycle) on three successive days to kill the soil microbes [36]. *Eupatorium adenophorum* leaves and *B. rapa* seeds were surface sterilized using sodium hypochlorite (0.3% v/v) for 15 min, followed by three washes in distilled water. To avoid contamination, all materials, tools, and surfaces used in the experiment were sterilized by one of three methods: soaking in 0.3% aqueous NaOCl, autoclaving for 60–180 min (121°C, 0.105 MPa), or spraying with 70% ETOH. All data were normally distributed. Three-way ANOVAs were conducted on biomass with leachate treatment, soil source, and sterilization as fixed factors.

### Allelochemical concentrations and stability in soil

*Eupatorium adenophorum* rhizosphere and non-rhizosphere soils were collected from the upper 10 cm of the profile at three randomly chosen plots in each site. There were two soil samples (rhizosphere and non-rhizosphere) for each plot at each site. The field soil was prepared by removing the fine roots and crumbling the soil until it passed through a 40 mesh sieve (0.45 mm). Forty grams of rhizosphere or non-rhizosphere soil from each plot were weighed and extracted with 200 ml absolute methanol for 24 h at 25°C immediately after collection. Thus, there were 18 soil samples in total for allelochemical analyses (3 sites × 3 plots × 2 samples). As two primary allelochemicals (9-Oxo-10,11-dehydroageraphorone and 9β-Hydroxyageraphorone) had been identified in *E. adenophorum* leachates [28], [29], we added 8 ml of leachate to another 40 g of non-rhizosphere soil from each plot, which was an equal proportion of the leachate to soil as was used in the soil and sterilization experiments described earlier (200 ml leachate: 1 kg soil). These samples were extracted instantly and another set of samples was extracted 24 h after leachate addition. Thus, there were 24 samples in total for analyses of leachate persistence (3 sites × 3 plots × 2 extraction times, and plus 3 replicates in sand × 2 extraction times). In all experiments, the extract was centrifuged at 4000 rpm for 40 min. The supernatant was further concentrated by rotary evaporator and resuspended in 2 ml of n-hexane. Excess debris was removed using a 0.45 µm syringe filter before GC-MS analyses.

A trace GC–MS spectrometer (ThermoQuest, San Jose, CA, USA) with a DB-5MS fused-silica capillary column (30 m×0.25 mm i.d., 0.25 µm film thickness) from J&W Scientific (Folsom, CA, USA) was used to analyze the soil extracts for the two main allelochemicals of *E. adenophorum*. The constant flow of carrier gas was 1.0 ml min^−1^ of high purity helium (99.999%). Data was collected with an Xcalib software data process system. The split/splitless injector was set to 250°C and 1 µl was injected with split (10). The ion source temperature was 200°C and the GC–MS interface was 250°C. The mass spectrometer was operated in the electron impact (EI) mode by full SCAN detection mode using 70 eV (35–600 U) and the dwell time (or scan time) for each SCAN transition was 0.5 s. The temperature of the column was initially 50°C, held for 5 min, then rose to 180°C at a rate of 15°C min^−1^, held for 3 min, and then rose to 290°C at a rate of 10°C min^−1^, held for 10 min.

## Results

### Soil experiment

**Figure 1:**
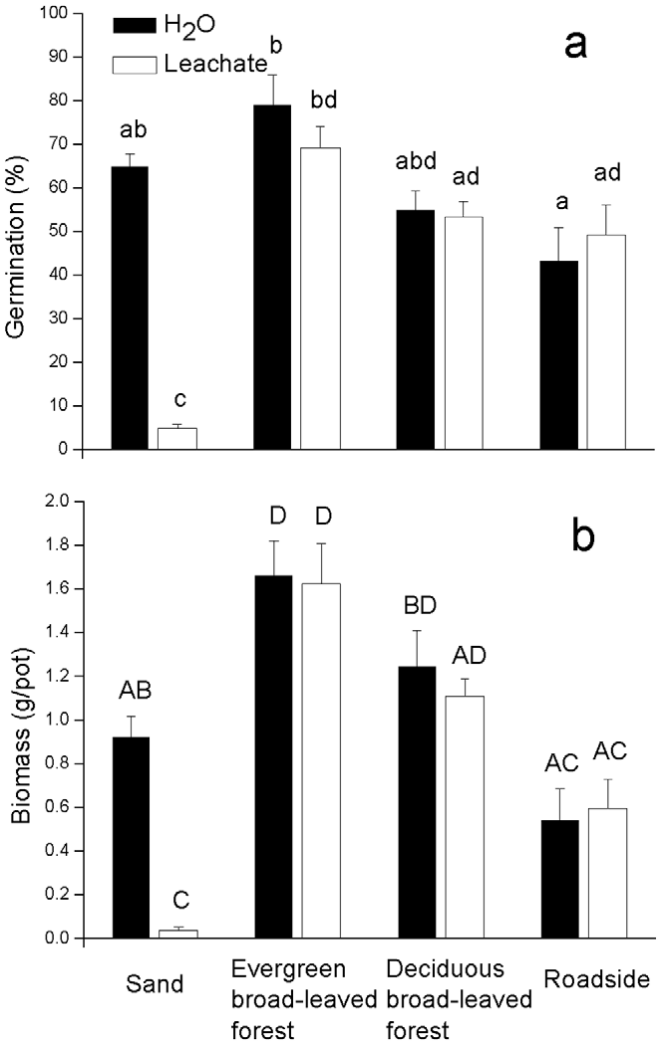
Effects of *E. adenophorum* leachate and soil source on a germination and b biomass of *Brassica rapa*. Error bars represent one standard error. For different soil types within either panel, different letters indicate significant differences (P<0.05) according to Tukey's Honest Significant Difference test. The key in the figure applies to both panels, a and b. See Table 2 for ANOVA results.

In the soil experiment, there were significant interactions between effects of *E. adenophorum* leachate and soil source (Table 2). *Eupatorium adenophorum* leachate added to sand dramatically reduced *B. rapa* seed germination (*c.* 92%) and seedling growth (*c.* 96%) (Fig. 1). In contrast, leachate had no effect on *B. rapa* in evergreen broad-leaved forest, deciduous broad-leaved forest and roadside soils (Fig. 1).

**Table 2:** ANOVA for the effects of *E. adenophorum* leachate and soil source on *B. rapa* germination and biomass.

Effect	df	MS	*F*	*P*
Germination				
Leachate	1,31	2167.014	19.897	<0.001
Soil	3,31	2179.977	20.016	<0.001
Leachate×soil	3,31	1768.866	16.241	<0.001
Biomass				
Leachate	1,31	0.513	7.491	0.011
Soil	3,31	2.392	34.960	<0.001
Leachate×soil	3,31	0.370	5.404	0.006

### Sterilization experiment

**Figure 2:**
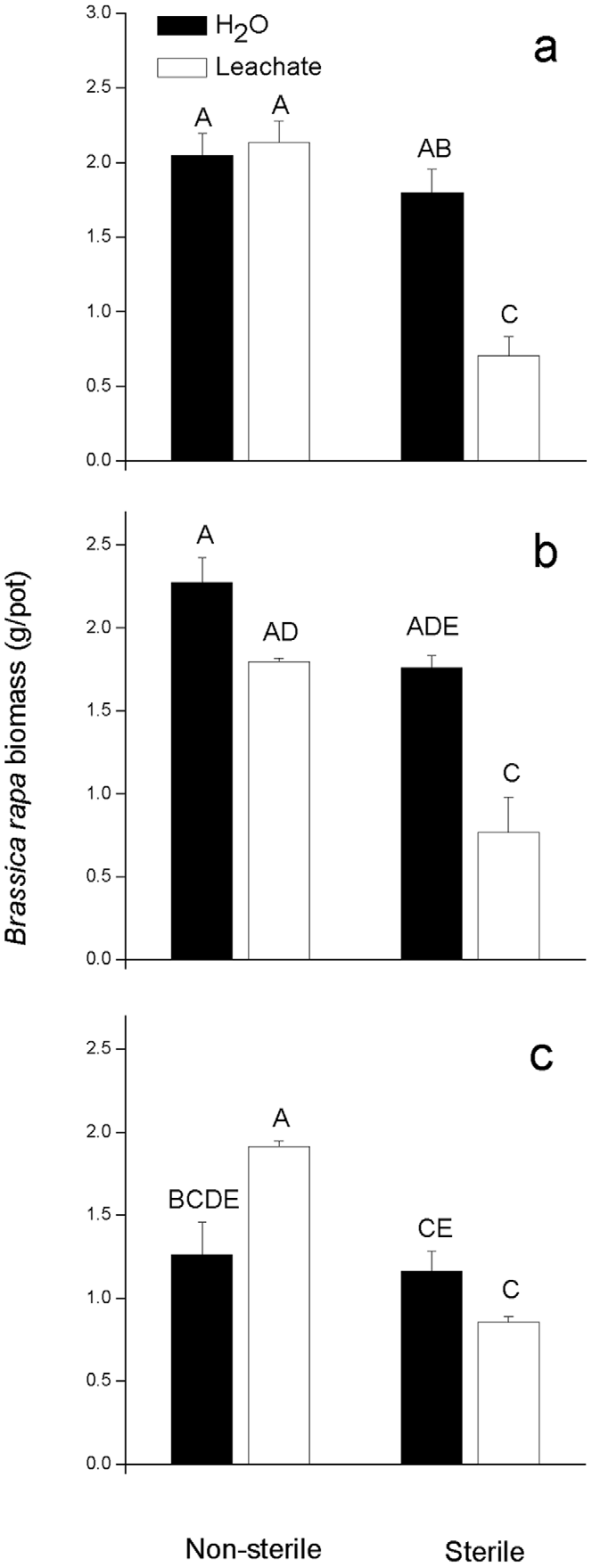
Differences in biomass of *B. rapa* plants treated with *E. adenophorum* leachate or distilled water in non-sterilized and sterilized soil collected from a evergreen broad-leaved forest, b deciduous broad-leaved forest and c roadside. Error bars represent one standard error. For all the treatments in this figure (all three panels together), different letters indicate significant differences (P<0.05) according to Tukey's HSD test. The key in the figure applies to all panels, a, b and c. See Table 3 for ANOVA results.

When the evergreen broad-leaved forest, deciduous broad-leaved forest and roadside soils were sterilized, however, responses of *B. rapa* to leachate treatment changed. *E. adenophorum* leachate had stronger negative effects on the growth of *B. rapa* seedlings in sterilized soil (Table 3, Fig. 2). In the evergreen broad-leaved forest soil, *E. adenophorum* leachate did not affect *B. rapa* growth in non-sterile soil, but decreased *B. rapa* biomass by 61% in sterile soil. In the deciduous broad-leaved forest soil, *E. adenophorum* leachate decreased *B. rapa* biomass to 79% of the control in non-sterile soil, but to only 44% of the control in sterile soil. Finally, in the roadside soil, *E. adenophorum* leachate increased *B. rapa* biomass by 51% in non-sterile soil, but reduced *B. rapa* biomass to 73% of the control in sterile soil.

**Table 3:** ANOVA for the effect of *E. adenophorum* leachate, soil source, and sterilization on *B. rapa* biomass.

Effect	df	MS	*F*	*P*
Leachate	1,47	2.254	37.289	<0.001
Soil	3,47	1.127	18.641	<0.001
Sterilization	1,47	4.791	79.262	<0.001
Leachate×soil	3,47	0.593	9.817	<0.001
Leachate×sterilization	1,47	1.759	29.102	<0.001
Soil×sterilization	3,47	0.581	9.621	<0.001
Leachate×soil×sterilization	3,47	0.271	4.491	0.007

### Allelochemical concentrations and stability in soil

**Figure 3:**
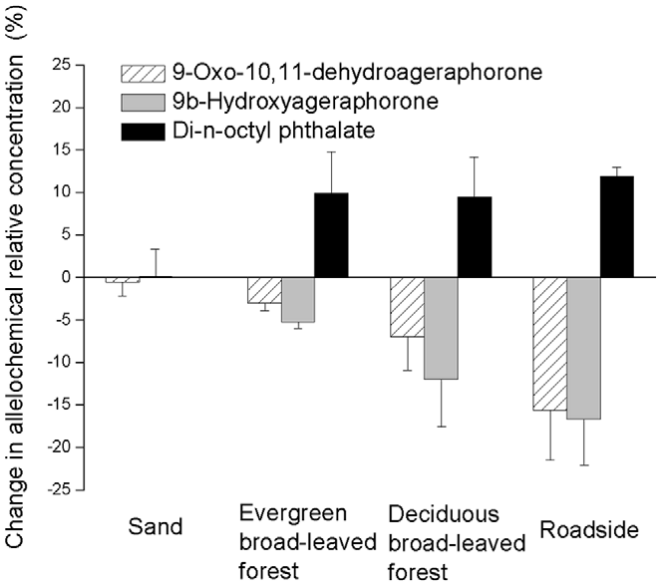
The change in relative concentration of two primary allelochemicals in *E. adenophorum* leachates (9-Oxo-10,11-dehydroageraphorone and 9β-Hydroxyageraphorone) and a potential degradation product (Di-n-octyl phthalate) in 24 h. Here the relative concentration refers to the proportion of the peak area in GC-MS chromatogram of each chemical. It is the relative content of that allelochemical in all the chemicals (Molecular Weight from 0–500) extracted from the leachate. The change in each allelochemical relative concentration equals to the proportion of this chemical after 24 h minus the one before 24 h. Error bars represent one standard error. One-way ANOVAs were conducted for each of the three chemicals. For 9-Oxo-10,11-dehydroageraphorone, *P* = 0.078; for 9β-Hydroxyageraphorone, *P* = 0.088; for Di-n-octyl phthalate, *P* = 0.142.

As other studies have already established that these are effective procedures for extracting and analyzing the two potential alleochemicals [27]–[29], we found that neither of the two main allelochemicals (9-Oxo-10,11-dehydroageraphorone or 9β-Hydroxyageraphorone) was detected in either rhizosphere or non-rhizosphere soils from any of the three field sites. However, when aqueous leachate of *E. adenophorum* leaves was added to soil and extracted immediately, both allelochemicals were detected by GC-MS. When the soils were incubated for 24 h after the leachates was added, relative concentrations of the two allelochemicals from all soils decreased substantially (Fig. 3). More GC-MS results suggested that the relative abundance of Di-n-octyl phthalate dramatically increased in all of the three soil types after 24 h of incubation (Fig. 3). In contrast, there was little difference in relative concentrations of the two allelochemicals before and after 24 h of incubation in sand culture. Stability of the two allelochemicals was greater in sand than in any of the three soils, and no new organic compounds became apparent in sand.

## Discussion

Our results demonstrate that soil biota could reduce allelopathic effects of *Eupatorium adenophorum*. Although a large number of studies have demonstrated phytotoxicity of *E. adenophorum* leachate *in vitro* and in sand cultures [25], [26], our experiments with field soils help to put these allelopathic effects into the context of natural soil. The soil experiment suggested that *E. adenophorum* leachate toxicity is greatly reduced in natural soil. *Brassica rapa*, a species previously reported to be sensitive to *E. adenophorum* leachate in laboratory bioassays [24]–[26], did not exhibit dramatic negative responses to *E. adenophorum* leachate in natural soils. Although the intensity of *E. adenophorum* invasion differed substantially among the three study sites, all three soils reduced the phytotoxic effects of the leachate (Fig. 1), suggesting that the ability of field soil to reduce allelopathic effects was not induced by *E. adenophorum* establishment.

In the soil sterilization experiment, *E. adenophorum* leachates were more toxic to *B. rapa* in sterilized soils (Fig. 2), suggesting that soil microbial communities were responsible for the lack of phytotoxicity in natural soils (Fig. 1). These results may explain the results of previous studies, which indicated that adding activated carbon to soil did not facilitate the growth of native plant species in competition with *E. adenophorum* and why *E. adenophorum* did not affect neighbor plants via allelopathy during the initial phases of invasion [34], [35]. However, the soil biota in the three soils did differ in their effects on leachate phytotoxicity in the sterilization experiment (although not in the soil experiment) (Fig. 2). The soil biota in the evergreen broad-leaved forest soil eliminated phytotoxicity entirely, whereas the soil biota in the deciduous broad-leaved forest soil only reduced phytotoxicity, and leachate treatment in the roadside soil promoted growth in the presence of soil biota (Fig. 2). Recently, Thorpe *et al.* [13] found that root exudates from the invasive weed *Centaurea maculosa* were allelopathic to native species in its invaded range but not in its native range. This lack of allelopathic effect in the native community may have been due to biological degradation of allelochemicals by native soil microbes, rather than, or in addition to, adaptation by native plants [8].

It is surprising that we did not detect either of the two primary allelochemicals in either rhizosphere or non-rhizosphere soils infested with *E. adenophorum*. Quantification and stability analyses of allelochemicals explained the reason for the disparity between previous studies of leachate phytotoxicity by laboratory bioassay and our experiments in soil. 9-Oxo-10,11-dehydroageraphorone and 9β-Hydroxyageraphorone decomposed by *c.* 3–20% within 24 h in soil but not in sand, while the relative abundance of another compound, Di-n-octyl phthalate, greatly increased concurrently, suggesting that soil microbes may transform 9-Oxo-10,11-dehydroageraphorone or 9β-Hydroxyageraphorone into Di-n-octyl phthalate. Zhang [27] demonstrated that Di-n-octyl phthalate occurs in the root exudates of *E. adenophorum*, but the biological activity of this compound is not clear. Soil microbes and *E. adenophorum* may contain the same or similar enzymes that catalyze a series of chemical reactions to make this change occur.

Actually, there are many factors that could influence the results of the soil experiment and the sterilization experiment, such as the composition of the leachate and the environmental conditions. Zhang [27] found that besides the two main allelochemicals, there are some other organic compounds in *E. adenophorum* leachate, which are possibly act alone or in combination to influence the growth of native plant species more or less even if they are not allelopathic. In different phenological stage, the composition and concentration of all kinds of chemicals including allelochemicals in *E. adenophorum* leaves are different, thus may cause a little difference in the leachates even if the extraction methods are the same. This can subsequently result in the different results in non-sterile soils between the soil experiment and the sterilization experiment. Also, the infiltration and spread efficiency can vary among field soil types or even the same type of soil in different seasons of the year (dry season or rain season). For example, the leachate which was poured into the soil may be diluted by soil water in more moist soils. Furthermore, the phytotoxicity of any chemical may vary with extraction methodology, analytical techniques and the vagaries of experimental application procedures. So it is not curious to find some instability of the data shown here.

Our findings from both manipulative greenhouse experiments and chemical analyses contribute to the growing body of evidence for the role of soil biota in plant interactions. Such complex effects depend on the presence of all interacting parties - the invader, native plants, and soil microbes. Soil microbial communities may regulate interactions between *E. adenophorum* and native species by breaking down allelochemicals excreted by the invader. These indirect effects in turn may influence plant community organization and structure and even ecosystem function, perhaps contributing to species coexistence and diversity. Soil microbes may be an important factor contributing to biotic resistance of native communities to *E. adenophorum* invasion, even though *E. adenophorum* is already one of the worst invaders in China. Also, the rapid degradation or transformation of *E. adenophorum* allelochemicals in soil has implications for restoration of cropland or rangeland infested with *E. adenophorum*. If Di-n-octyl phthalate is not toxic to native species, then restoration with native plants should be feasible almost immediately following *E. adenophorum* removal.

Our results also suggest that effects of soil biota on allelopathy may depend on how the allelochemicals are delivered. If leachates containing phytotoxins fall directly on a seed or on target plant tissues, or at least reach the target quickly, then soil biota may have little effect on allelopathic interactions. Similarly, if phytotoxins in root exudates contact a competitor's root immediately because the roots are in contact, then soil biota may have little effect. In contrast, if allelochemicals must travel through a lot of soil, or have a long residence time in soil, to reach a competitor, then soil biota may have substantial effects on allelopathic interactions.

In summary, our results demonstrate that allelopathic compounds can be degraded by soil biota in natural settings, and that soil microbial communities have the potential to ameliorate or eliminate the phytotoxic effects of allelochemicals excreted by E. *adenophorum*. Similar experimental phenomena were observed recently in the study of allelochemical degradation or mineralization in soil or decomposition by microbes, such as the research on allelopathic secondary metabolites excreted by *Alliaria petiolata* [37], a potent plant growth inhibitor *m*-Tyrosine [38]–[39], phenolic allelochemicals [40] and the allelochemical sorgoleone in soil [41]. Those results would provide a significant step toward understanding the degradation processes of allelochemicals in the natural environment, and illustrate how lab experiments suggesting allelopathy may not hold up in more natural settings. Although our study did not include a biogeographic comparison of *E. adenophorum* allelopathic effects on naïve, native plants and plants from *E. adenophorum*'s native range, our results provide evidence in a way against the novel weapons hypothesis for *E. adenophorum* invasion by demonstrating that *E. adenophorum* has no measurable allelopathic effects on a native plant species in the invaded range.
